# Sjogren's Antibodies and Neonatal Lupus: A Scoping Review

**DOI:** 10.7759/cureus.62528

**Published:** 2024-06-17

**Authors:** Deepika Nagliya, Courteney Castellano, Michelle L Demory, Marc M Kesselman

**Affiliations:** 1 Osteopathic Medicine, Nova Southeastern University Dr. Kiran C. Patel College of Osteopathic Medicine, Davie, USA; 2 Immunology, Nova Southeastern University Dr. Kiran C. Patel College of Allopathic Medicine, Davie, USA; 3 Rheumatology, Nova Southeastern University Dr. Kiran C. Patel College of Osteopathic Medicine, Davie, USA

**Keywords:** sjogren syndrome antibodies or ssa ssb, congenital heart block, fetal echocardiogram sjogren syndrome, sjogren syndrome prenatal, stop bloq, neonatal lupus, sjogren syndrome and pregnancy, hydroxychloroquine sjogren syndrome

## Abstract

Sjogren's syndrome (SS) is an autoimmune disease characterized by inflammation of exocrine glands. The disorder predominantly affects middle-aged women. Autoantibodies, including anti-SS-A/Ro and anti-SS-B/La antibodies, are present in most cases of SS. These antibodies can cross the placenta and likely play a role in pregnancy complications as well as the development of neonatal lupus, resulting in congenital heart block (CHB). It is essential to monitor the fetus for CHB during pregnancy. In particular, screening with echocardiography and monitoring heart rate at home are recommended practices. Regarding medical management, hydroxychloroquine and glucocorticoids have shown promise in reducing cardiac manifestations, but further research is needed to elucidate their longer term efficacy and safety. This scoping review analyzes literature from 2001 to 2024, focusing on pregnancy outcomes among women with SS, clinical manifestations of neonatal lupus, the role of anti-SS-A/Ro and anti-SS-B/La antibodies in the development of neonatal lupus and CHB, and emphasizes the need for future research efforts to refine treatment protocols and enhance clinical care strategies for pregnant women with SS.

## Introduction and background

Healthy pregnancies, approximately 88% of all pregnancies, require basic care. This includes regular prenatal checkups, nutrition and vaccination counseling, screening of gestational diabetes, and other conditions. High-risk pregnancies (which include the presence of comorbid hypertension, diabetes, infectious diseases, and/or autoimmune diseases), approximately 12% of all pregnancies, require additional and specific interventions.

Sjogren's syndrome (SS) is an autoimmune disease, with a global estimated prevalence of 0.1%-8.4% in different studies [1]. SS predominantly affects middle-aged women, with a peak age of 56 [2]. It is marked by a chronic inflammatory infiltrate of activated T and B cells into exocrine glands, commonly affecting the lacrimal and salivary glands [1]. This infiltration can reduce the glands' secretary function, giving rise to the classic symptoms of dry eyes and mouth [3].

SS can present alone as primary SS (pSS) or in association with an underlying connective tissue disease as secondary SS (sSS). The most common underlying connective tissue disorders in sSS include rheumatoid arthritis or systemic lupus erythematosus (SLE) [2]. Autoantibodies are present in most SS cases including anti-SS-A/Ro and anti-SS-B/La antibodies. These antibodies bind to Ro and La antigens, respectively, on the cell surfaces of lacrimal and salivary glands, triggering an immune response, leading to tissue damage in SS. These antibodies can cross the placenta starting at 12 weeks of gestation and can pose a risk to fetal tissue. Consequently, these self-reactive antibodies appear to be ultimately responsible for pregnancy complications in women with SS, including the clinical manifestations of neonatal lupus [4].

Pregnant women with underlying autoimmune conditions tend to have complications in their pregnancies compared to women without the condition. A recent U.S. investigation of 1.5 million births found a 4.8-fold higher rate of severe maternal complications among women with preexisting diseases [5]. The overall influence of autoimmune conditions on the course and outcome of pregnancy is dependent on factors including type of maternal disease, antibody profile, activity level, and drug treatment. Well-known fetal outcomes of pregnancies complicated by SS include neonatal lupus and congenital heart block (CHB) [4]. Other clinical manifestations linked with anti-SS-A/Ro and anti-SS-B/La antibodies include transient skin rash in neonates, liver abnormalities, and thrombocytopenia [4].

## Review

Methods

**Figure 1:**
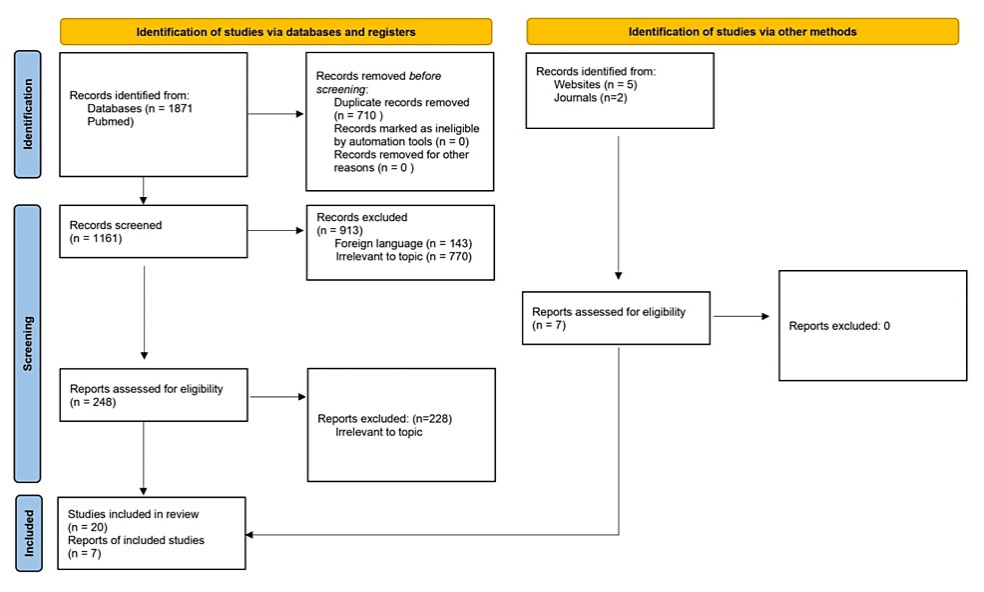
Preferred Reporting Items for Systematic Reviews and Meta-Analyses flow diagram

A scoping review of the literature was conducted using Pubmed and Embase. During the research, the keywords used to search articles were “Sjogren syndrome and pregnancy,” “neonatal lupus,” “Sjogren syndrome antibodies” OR “SSa SSb,” “Congenital heart block,” “Sjogren syndrome prenatal,” “fetal echocardiogram Sjogren syndrome,” “STOP BLOQ,” and “hydroxychloroquine Sjogren syndrome.” UpToDate was searched with emphasis on the keywords “neonatal lupus” and “Sjogren syndrome prenatal.” The manuscripts in this scoping review were peer-reviewed and published in English between 2000 and 2024 (inclusion criteria). There were no limitations on race, age, gender, or ethnicity. Excluded studies included articles published in languages other than English, duplicates, and study abstracts. Our preliminary search yielded 1,871 articles. After assessing articles for eligibility, 27 articles were included in the final analysis. This process can be visualized in the Preferred Reporting Items for Systematic Reviews and Meta-Analyses flow diagram (Figure 1).

Pregnancy outcomes with SS

Pregnant women with autoimmune diseases have a 4.8-fold higher rate of severe maternal complications during pregnancy compared to those without these conditions [5]. Factors that have been demonstrated to be involved in predicting the fetal outcome among women with autoimmune diseases include the status of maternal disease activity, severity of organ damage, antibody profile, and specific drug treatment [6]. Commonly, it is the activity of the disease that correlates directly with the risk of fetal outcomes, especially poor fetal outcomes [6].

Recent studies have demonstrated that SS may be associated with adverse pregnancy outcomes such as preterm delivery and miscarriage. A meta-analysis evaluating the effect of SS on adverse pregnancy outcomes found that women with SS have shorter gestation times, a higher incidence of preterm delivery, lower birth weight percentiles, and a greater proportion of small-for-gestational-age infants, as compared to their healthy counterparts [7]. A significantly lower mean neonatal birth weight (<2,500 g) was found among pSS pregnancies compared to healthy controls. In addition, pSS pregnancies were shown to be complicated by intrauterine atrioventricular (AV) block [7]. Furthermore, a systematic review and meta-analysis found that compared with healthy pregnancies, patients with pSS had a significantly higher chance of neonatal deaths [8]. The presence of pregnancy comorbidities, including pregnancy hypertension and preeclampsia/eclampsia, is significantly higher among pregnant women with SS compared with those without SS [9]. Pregnancy complications like postpartum deep vein thrombosis have a higher incidence in pregnancies complicated by SS [9]. Furthermore, symptoms of SS are typically worse during pregnancy. The severity of the disease is typically most notable in the postpartum period due to the complications of pulmonary hypertension [6].

Well-known fetal outcomes associated with pregnant women with SS include neonatal lupus and CHB [4]. Neonatal transient skin rash, liver abnormalities, and thrombocytopenia are some of the other clinical manifestations associated with anti-SS-A/Ro and anti-SS-B/La antibodies [4]. Patients with anti-SS-A/Ro antibodies have an increased risk of having a child with neonatal lupus. Infants of women with SS are also more likely to have intrauterine growth restriction (IUGR) and congenital malformations [7].

Neonatal lupus

Neonatal lupus is a rare autoimmune disease manifested clinically by cutaneous lupus lesions and/or CHB. Skin lesions of neonatal lupus are rarely present at birth and typically appear within the first few days or weeks of age. The lesions can occur anywhere on the skin but most commonly on the face and scalp [10]. The lesions appear as annular erythematous plaques typically associated with central clearing and occasionally with scaling. The lesions resolve spontaneously after a few weeks or months [10].

Neonatal lupus is associated with three types of hepatobiliary disease: liver failure at birth or in utero, transient conjugated hyperbilirubinemia in infants, and transient transaminase elevations in infants [11]. The incidence of these diseases is approximately 10%. The transient conjugated hyperbilirubinemia and transaminase elevations resolve spontaneously with no long-term complications [10]. Hematologic diseases, such as transient thrombocytopenia, have been reported in neonatal lupus cases, with an incidence of 10% of cases [10]. Anemia and neutropenia have also been reported less frequently.

Later in life, children with neonatal lupus have an increased risk of developing autoimmune diseases, especially if they have a family history of autoimmune diseases. In a long-term follow-up study of 49 children with neonatal lupus and 45 of their unaffected siblings, six children with definite rheumatic/autoimmune diseases were identified [12]. Of these six children, two developed juvenile rheumatoid arthritis, one experienced Hashimoto thyroiditis, another experienced psoriasis and iritis, one resulted in diabetes mellitus and psoriasis, and one faced congenital hypothyroidism and nephrotic syndrome [13]. As such, children with neonatal lupus require close follow-up.

Mechanisms of disease activity: anti-SS-A/Ro and anti-SS-B/La antibodies

Anti-SS-A/Ro and anti-SS-B/La antibodies are important markers of SS disease activity. Autoantibodies targeting antigen type A bind to two cellular proteins after apoptosis: Ro52/52 kD, present in both the nucleus and cytoplasm, and Ro60/60 kD, found in the nucleus and nucleolus. Autoantibodies directed against antigen type B specifically bind to a 47 kD protein between the nucleus and the cytoplasm. These antibodies recognize specific cellular proteins released during cell death, which the immune system can then target. These antibodies are associated with earlier onset of the disease, longer disease duration, more severe dysfunction of the exocrine glands, recurrent parotid gland swelling, and increased severity of the lymphocytic infiltrates in the minor salivary glands [14].

In one study by Robbins et al., the researchers analyzed the serum of 13,032 patients for anti-SS-A/Ro antibodies. The investigators found that SLE was prevalent in patients with anti-Ro60-positive and anti-Ro52-negative antibodies, whereas in patients with both anti-Ro60- and anti-Ro52-positive antibodies, SS was the predominant diagnosis. Further research is needed to identify the specific epitope responsible for neonatal lupus, but some animal models have indicated an increased role of anti-Ro52 in disease development. Meanwhile, it has been shown that mothers with anti-SS-A/Ro and anti-SS-B/La antibodies had an increased risk of heart complications in neonatal lupus, with the most clinically significant manifestation associated with positive anti-Ro52 being the development of CHB.

In another study by Salomonsson et al., the researchers evaluated the serological profile of mothers of infants with CHB. The investigators found that 95% of mothers had a positive anti-Ro52 antibody test [15]. In addition, positive anti-Ro52 animal models with CHB also develop AV block. Additional research efforts by Jaeggi et al. and Buyon et al. demonstrated that mothers with low antibody titers had a lower risk of giving birth to newborns with lupus. The researchers of both studies determined that none of the fetuses had cardiological complications in the form of heart block [15]. The results suggest that anti-SS-A/Ro antibodies, particularly anti-Ro52, are strongly associated with the development of CHB in neonatal lupus, emphasizing the need for further research on specific epitopes and mechanisms.

Congenital heart block

CHB is the most severe outcome of SS pregnancies and has an estimated incidence between 1% and 5% in all infants born to women with anti-SS-A/Ro antibodies [16]. In mothers with a previous child affected, the recurrence rate is 15%-20% [16]. CHB may begin in utero in the second or third trimester and can be detected shortly after birth. CHB presents as either a first-, second-, or third-degree heart block. Third-degree AV block is irreversible and is the most severe manifestation of neonatal lupus due to its high morbidity and mortality rate [5]. In rare cases, heart block begins as a first- or second-degree block and then progresses to a third-degree block [10]. This heart block occurs without structural heart abnormalities [10].

CHB is frequently detected in utero via prenatal ultrasound between 18 and 24 weeks of gestation [16]. The key sign associated with CHB is related to the ventricular rate (range between 30 and 100 beats/min). Fetal or neonatal death correlates with a ventricular rate less than or equal to 55 bp [16].

Commonly, the treatment of CHB requires pacemaker implantation. Pacemaker therapy in pediatric patients is determined based on guidelines from the American Heart Association (AHA) and the American College of Cardiology. Newborns may require a pacemaker if they exhibit indicators such as a ventricular rate below 55 beats/minute, cardiovascular issues with impaired heart function, or a prolonged QT interval [13]. Some patients with CHB may develop dilated cardiomyopathy, with an incidence of approximately 6% [5]. Treatment requires cardiac transplantation. In a multi-institutional study, the age at cardiac transplantation ranged from nine months to ten years [17].

The exact underlying mechanisms associated with CHB in utero have been linked to anti-SS-A/Ro and anti-SS-B/La antibodies, and result in neonatal transient skin rash, liver abnormalities, and thrombocytopenia. These antibodies deposit in the AV node cardiac tissue. In vitro and animal model studies of CHB highlight that maternal anti-Ro52 antibodies cross-react with fetal cardiac molecules involved in calcium regulation, like ion channels type L and T, triggering cardiac conduction issues. Prolonged disturbances in calcium regulation may induce fetal heart apoptosis, exposing Ro and La autoantigens to maternal antibodies, leading to inflammation and fibrosis in the fetal heart, and resulting in permanent damage and complete AV block [18].

Treatment of CHB

Hydroxychloroquine (HCQ) is an antimalarial drug used in multiple connective tissue conditions and can be continued during pregnancy because of its benign adverse event profile, posing a limited risk to the mother and neonate. Data suggest that HCQ lowers cardiac manifestation of neonatal lupus. In one study by Izmirly et al., the investigators examined 257 pregnant women from neonatal lupus registries in the United States, France, and the United Kingdom. The patients included in the study were anti-SS-A/Ro positive and had a prior history of giving birth to a child with cardiac neonatal lupus. Among the 257 patients, 40 were exposed to HCQ, and 217 were unexposed to HCQ. The recurrence rate of cardiac manifestations in fetuses exposed to HCQ was 7.5% compared to 21.2% in the unexposed group (p = 0.050). The study also revealed a significantly lower case fatality rate in the exposed group compared to the unexposed group [19]. These findings suggest that HCQ exposure may be associated with a reduced recurrence rate of cardiac manifestations and a lower case fatality rate in fetuses of anti-SS-A/Ro-positive pregnant women with a history of cardiac neonatal lupus.

The period of emergent complete AV block, which includes the first-degree AV block and the second-degree AV block, might represent the only opportunity for anti-inflammatory intervention to restore normal rhythm [20]. A study conducted by Friedman et al. prospectively evaluated fetuses with autoimmune-associated CHB as part of the PR Interval and Dexamethasone Evaluation study.

The study included 40 mothers with fetuses diagnosed with CHB exhibiting varied maternal health statuses, including autoimmune disorders such as SS and SLE. All mothers with a previously affected child were included in the dexamethasone treatment group. Thirty mothers received dexamethasone treatment, while 10 did not. Out of the 10 mothers with no dexamethasone treatment, nine exhibited third-degree block and one first-degree block. Consistent with prior research findings, no instances of the third-degree block reversal were observed in either group. Among the six fetuses in the second-degree heart block subgroup treated with dexamethasone, only one progressed to the third-degree block. Dexamethasone treatment showed no significant difference in median anti-SSA/Ro antibody titers and similar fetal ventricular rates at CHB diagnosis. Meanwhile, the dexamethasone group experienced 20% fetal deaths and a higher rate of prematurity. Pacemaker use was observed in 43% of cases by two years, with no significant difference between dexamethasone and non-dexamethasone groups.

Overall, dexamethasone treatment correlated with lower gestational age at birth and comparable pacemaker utilization but was associated with higher prematurity rates and fetal mortality [21]. A systematic review and meta-analysis of five observational studies involving 71 fetuses with second-degree AV block revealed that the rate of progression to complete AV block was 52% in fetuses treated with fluorinated glucocorticoids and 73% in those not treated with fluorinated glucocorticoids. The rate of regression from second-degree AV block to first-degree, intermittent first/second-degree, or normal sinus rhythm was 25% in treated fetuses and 23% in untreated fetuses. In addition, the rate of complete regression to normal sinus rhythm was 21% in treated fetuses compared to 9% in untreated fetuses [22]. However, the decision to administer steroids to a fetus with the first-degree block is still not well-supported by evidence, as the first-degree block does not always progress to more severe forms of heart block.

Further implications

Antenatal Management

Fetal echocardiography is an essential screening tool for detecting CHB, a serious complication of neonatal lupus. Arrhythmias are usually diagnosed 18 weeks after pregnancy, but a few studies report diagnosis at 16-17 weeks. Meanwhile, fewer than 20% of cases are detected after 26 weeks of gestation. IgG autoantibodies start to pass into the fetal bloodstream during pregnancy, typically around the 12th week of gestation [15]. The AHA recommends screening at 16 weeks of gestation and then every week or every other week until 28 weeks of gestation in women with positive anti-SS-A/Ro and anti-SS-B/La autoantibodies and for women who have a history of a previously affected child at least every week during the same time frame (16-20 weeks). Studies have shown that 2% of AV block cases are diagnosed in the neonatal period after birth, which is why it is necessary to observe newborns during their first month of life. Anti-SS-A/Ro antibodies account for 80%-95% of reported cases of CHB in fetuses and neonates [23]. Buyon et al. recommend testing for these antibodies in mothers of neonates diagnosed with heart block and no causal structural abnormalities.

As part of the "Surveillance and Treatment to Prevent Fetal AV Block Likely to Occur Quickly (STOP BLOQ)" trial, Bettina F. Cuneo, M.D., of the University of Arizona in Tucson, Jill P. Buyon, M.D., of New York University (NYU), and colleagues found that regular monitoring of fetal heart rate daily was most beneficial for pregnant women exhibiting elevated levels of anti-SS-A/Ro antibodies in their blood. Among 413 pregnant women who participated in the study and had anti-SSA/Ro antibodies, 45 Doppler recordings were flagged as potentially abnormal out of over 30,000 sent, which were referred for immediate echocardiogram testing [24]. The researchers found that 10 of the 45 cases of heart block were confirmed, including seven being identified as early-stage heart block and promptly treated. Six of the ten occurred in pregnant women with notably elevated levels of anti-SS-A/Ro antibodies in their blood [19]. The study concluded that at-home fetal heart rate monitoring is a cost-effective method that allows early detection and improves the child’s prognosis.

In asymptomatic mothers, maternal testing for anti-SS-A/Ro and anti-SS-B/La should be done if there is a diagnosis of heart block in the fetus/postnatally, as neonatal lupus in a fetus can be the first sign that the mother has anti-SS-A/Ro and anti-SS-B/La antibodies [23]. Rivera et al. studied 321 mothers from the Neonatal Lupus Registry and found that half of the 321 asymptomatic women developed autoimmune diseases. Among the 26 affected, diagnoses included undifferentiated connective tissue disease, SS, and SLE. The probability of developing SLE after giving birth to a neonatal lupus erythematosus child was 18.6%, while for SS, it was 27.9%. Patients with positive anti-SS-A/Ro and anti-SS-B/La antibodies had a higher disease risk [15]. In another study, Martinez-Sanchez et al. evaluated 40 SS-A-positive mothers who gave birth to children with symptoms of neonatal lupus. Among these mothers, the researchers found that 40% were diagnosed with SLE, while 37.5% were found to have SS. This warrants further investigation into the importance of monitoring asymptomatic mothers for the development of systemic autoimmune diseases, which is crucial for early detection and intervention for both maternal and fetal health. Testing for antinuclear antibodies is further implicated for every pregnant woman to enable early treatment with HCQ or intravenous immunoglobulin, which has shown effectiveness in preventing CHB [25].

Medications

Studies involving the administration of 400 mg of HCQ daily beginning between the sixth and tenth weeks of pregnancy have demonstrated a reduced likelihood of neonatal cardiac lupus, particularly among women with a prior history of an affected infant. Several studies demonstrate that preventative treatment is not recommended in mothers without SS, primigravid mothers who are positive for anti-SS-A/Ro, or those with a prior history of noncardiac lupus.

Limited and conflicting data exist about the efficacy of steroids in neonatal lupus congenital AV block. While some studies have shown that steroid administration during pregnancy might prevent inflammatory damage to the fetal cardiac conduction system, mediated by maternal anti-SS-A/Ro and anti-SS-B/La autoantibodies, causing heart block, it is essential to consider the associated risks to both mother and fetus. These risks include hypertension, avascular necrosis, insulin resistance, and gestational diabetes for the mother, as well as oligohydramnios and growth restriction for the infant.

Several studies have examined the effectiveness of fluorinated glucocorticoids in treating first- and second-degree heart block detected prenatally, but the findings are inconsistent. Buyon recommends since first-degree AV block does not progress to advanced heart blocks; observation is reasonable. She further suggests for second-degree AV block, administering fluorinated glucocorticoids (i.e., oral dexamethasone 4-8 mg/day or betamethasone equivalent) as soon as possible upon detection [26]. Dexamethasone 2 mg/day is typically continued until the end of pregnancy, even if the heart block regresses, with tapering of therapy [26]. Evidence suggests fluorinated glucocorticoids have not shown a reversal of third-degree block and do not improve survival or prevent extranodal disease. As such, treating in utero third-degree block without myocarditis or cardiomyopathy signs with glucocorticoids is not recommended.

Preconception

SS affects 0.06% of the world’s population, with women comprising over 90% of those affected [24]. It has been shown that 10% of individuals with dry eye diseases also have SS [27]. Meanwhile, a significant portion remains undiagnosed, and there is a median delay in diagnosis of approximately 10 years [27]. Thus, further investigation should be conducted to determine whether women with dry eyes and mouths who are planning pregnancy should be screened for SS to improve the child’s prognosis.

## Conclusions

Overall, SS in pregnancy is responsible for preterm births, increased risk of spontaneous abortions, and lower birth weight percentile. Fetal outcomes of pregnancies complicated by SS include well-known complications, neonatal lupus, and CHB, as well as IUGR and congenital malformations. Maternal autoantibodies, anti-SS-A/Ro and anti-SS-B/La, play a role in the pathogenesis of CHB by binding to fetal cardiac tissue and inducing cardiac conduction disturbances. Jill P. Buyon, M.D., of NYU, and colleagues recommend that early detection and management of these antibodies, along with close fetal monitoring through techniques like fetal echocardiography and at-home fetal heart rate monitoring, are essential in improving pregnancy outcomes. One common approach to monitoring fetal heart rates at home is using a handheld Doppler device, which allows expecting mothers to listen to and measure their baby's heartbeat. These devices are widely available and relatively easy to use, typically involving applying a small amount of ultrasound gel to the abdomen and moving the Doppler probe around until the heartbeat is detected. The studies highlight that echocardiography screening can be reduced in the absence of an AV block diagnosis. Medications like HCQ and glucocorticoids may offer therapeutic options, but their efficacy and safety need further investigation. In addition, experts recommend that preconception screening for SS and regular monitoring of asymptomatic mothers with a history of fetal complications are vital for timely intervention and prevention of adverse outcomes. Overall, interdisciplinary care involving obstetricians, rheumatologists, and other specialists is crucial in providing comprehensive care to pregnant women with SS. Further research is needed to enhance our understanding of the pathogenesis underlying SS-related pregnancy complications and to develop more effective management strategies.
